# Adrenal Insufficiency Following Prolonged Administration of Ultra-High Topical Steroid: A Case of Refractory Dermatitis

**DOI:** 10.7759/cureus.37967

**Published:** 2023-04-22

**Authors:** Tsunetaka Kijima, Naohide Shimada, Naoya Ishida, Shingo Yamagata, Testuya Makiishi

**Affiliations:** 1 Department of General Medicine, Faculty of Medicine, Oda Training Center of General Practice, Oda Municipal Hospital, Shimane University, Oda, JPN; 2 Department of General Medicine, Faculty of Medicine, Shimane University, Izumo, JPN; 3 Department of General Medicine, Oda Municipal Hospital, Oda, JPN; 4 Department of Neurology, Oda City Hospital, Oda, JPN

**Keywords:** adverse drug reaction, topical steroid, dupilumab, adrenal insufficiency, new symptoms

## Abstract

Replacement of a usual medication with a remarkably effective medication might result in a dramatic improvement for a specific disease. However, an abrupt change in medication might bring about other challenges. Herein, we report the case of an 84-year-old man who developed severe hyponatremia after the abrupt discontinuation of prolonged ultra-high topical steroid use. At the time of visiting the emergency department, he had been treating chronic eczema with the medication dupilumab for three months. We initially considered this newly started medication as the cause of the problem. However, dupilumab has not been reported to be connected to any electrolyte or endocrine disorder (e.g., syndrome of inappropriate anti-diuretic hormone secretion), and severe hyponatremia did not improve by the administration of high volume of NaCl. Thus, we reconsidered alternative causes for this hyponatremia and checked the patient’s history of medication. He had been prescribed clobetasol propionate 0.05% by the dermatologist until one month before arriving at the emergency department. In addition, he had completely stopped using topical steroids for the last two weeks because his dermal condition had substantially improved. His cortisol level was low, substantiating a diagnosis of adrenal insufficiency. Hydrocortisone administration improved both hyponatremia and his symptoms. Therefore, when a patient with newly administered medication presents with new symptoms, we recommend that differential diagnosis include a medical review of the patient’s last three months of medication and the conditions of use including how the topical agents were used.

## Introduction

Topical corticosteroids are the most commonly used drugs for inflammatory skin diseases, as they do not cause adverse side effects when appropriately used. Local and systemic side effects are reported only when excessive doses are administered. Atrophy, acne, rosacea, and purpura have been reported to be the most common local side effects [1, 2]. Though systemic side effects are much less common than local ones, hyperglycemia, glaucoma, adrenal insufficiency, and Cushing syndrome may also occur [1]. Dupilumab is a new medication used to treat moderate-to-severe atopic dermatitis (eczema) [3] and can improve dermatitis that is inadequately controlled by topical corticosteroids [3]. Newly emergent effective medication might result in previously used medical treatment becoming unnecessary. Herein, we report the case of an 84-year-old man who developed adrenal insufficiency owing to the abrupt discontinuation of a high dose of corticosteroids after dupilumab administration.

## Case presentation

An 84-year-old man was brought to our emergency department owing to a recent episode of seizure and loss of consciousness that lasted for a few minutes.

He had initially visited his primary care physician because he had diarrhea, fatigue, and loss of appetite. He was initially waiting in his car after undergoing a coronavirus disease 2019 antigen test in front of the medical office and presented a transient bilateral tonic convulsion when talking to the physician. He regained consciousness within a few minutes and was referred to our hospital. He did not undergo any change in consciousness or neurological abnormality at the emergency department. His blood pressure was slightly high (155/80 mmHg), and his other vital signs were normal. No abnormality was found during physical examination.

The patient’s past medical history included hypertension, duodenal ulcer, gastroesophageal reflux disease, benign hypertrophic prostate, and chronic eczema. His current list of medications included azilsartan, amlodipine, vonoprazan fumarate, mosapride citrate hydrate, rebamipide, silodosin, dutasteride, and distigmine bromide. Furthermore, he takes subcutaneous dupilumab for refractory eczema; this medication had been initiated three months prior.

Laboratory investigations showed the following: creatinine phosphokinase 984 U/L, blood urea nitrogen 9.2 mg/dL, creatinine 0.77 mg/dl, Na 114 mmol/L, Cl 86 mml/L, K 3.9 mmol/L, white blood cell count 4850/μL, hemoglobin 13.3 g/dL, and platelet count 157000/μL (Table 1).

**Table 1:** Laboratory data WBC: White Blood Cell count, RBC: Red Blood Cell count, Hb: Hemoglobin, PLT: Platelet count, Neut: Neutrophil, Lymph: Lymphocyte, Eosino: Eosinophil, TP: Total Protein, Alb: Albumin, CK: Creatine Kinase, BUN: Blood Urea Nitrogen, Cre: Creatinine, TSH: Thyroid Stimulating Hormone, FT4: Free Thyroxine 4, ACTH: Adrenocorticotropic Hormone, AVP: Arginine Vasopressin, AST: Aspartate Aminotransferase, ALT: Alanine Aminotransferase, LDH: Lactate Dehydrogenase, UA: Uric Acid, CRP: C-Reactive Protein.

Complete blood count			Blood Chemistry				
WBC	4.85 × 10^3^ /μl		TP	6.7 g/dl		K	3.9 mEq/l
RBC	433 × 10^4^ /μl		Alb	3.9 g/dl		Ca	8.5 mEq/l
Hb	13.3 g/dl		AST	53 IU/l		Glucose	112 mEq/l
PLT	15.7 × 10^3^ /μl		ALT	35 IU/l		CRP	0.04 mg/dl
Neut	16.9 × 10^2^ /μl		LDH	416 IU/l		TSH	1.85 μIU/ml
Lymph	24.3 × 10^2^ /μl		CK	984 IU/l		FT4	0.94 ng/dl
Eosino	2.3 × 10^2^ /μl		BUN	29.2 mg/dl		ACTH	43.0 pg/ml
Urinalysis			Cre	0.77 mg/dl		Cortisol	6.66 μg/dL
Protein	negative		UA	2.9 mg/dl		Aldosterone	4.3 pg/ml
Blood	negative		Na	114 mEq/l		Plasma renin activity	2.0 ng/ml/hr
Specific gravity	1.007		Cl	83 mEq/l		AVP	0.7 pg/mL

Head computed tomography did not show any abnormality. Transient convulsion was thought to be due to hyponatremia. Initially, syndrome of inappropriate secretion of anti-diuretic hormone (SIADH) was thought to underlie this condition. We screened the patient for pneumonia and medications being administered. No pneumonia was detected. As for medication, the patient had initiated the administration of a new drug, dupilumab, during the prior three months. This drug had no corresponding reports regarding SIADH. In addition, the patient claimed that his dermal condition had readily improved due to the administration of dupilumab. Therefore, we checked his medication history in more detail. He had previously used the ultra-highly rated topical corticosteroid clobetasol propionate 0.05% for the treatment of dermatitis, which had been prescribed at a dosage of 100 g for 2 weeks, for more than 4 months prior. Dupilumab had been started three months prior, simultaneously with clobetasol propionate, the amount of which had been decreased gradually. Though clobetasol propionate was being prescribed until one month before we treated him, the patient claimed that any use of the topical steroid had been completely discontinued two weeks before we examined him due to his good dermal condition.

**Figure 1:**
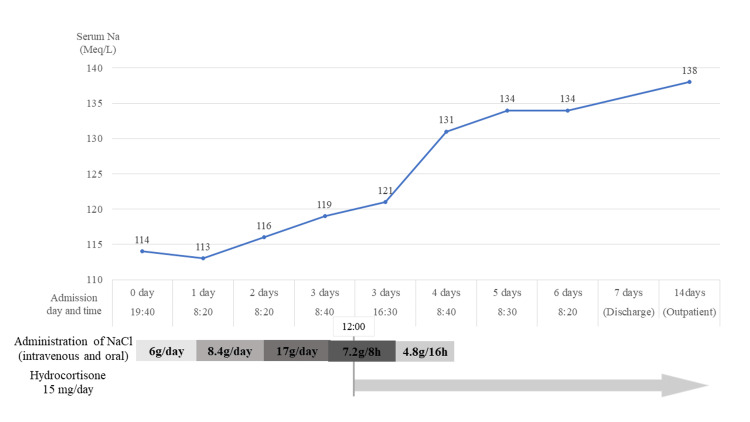
Process for serum Na levels and treatment

Although we administered a high dose of hypertonic saline (1.6% NaCl) and oral NaCl (3g/day), there was no resultant improvement in serum Na levels (Figure 1). After we prescribed hydrocortisone 15 mg/day for suspected adrenal insufficiency, the patient’s serum Na level quickly increased. Thus, we discontinued the NaCl administration. However, his serum cortisol level was found to be low (6.66 μg/dL), and the anti-diuretic hormone arginine vasopressin (AVP) level was low. We checked his abdominal CT findings, indicating no adrenal gland tumor, calcification, or swelling. Any tumor or swelling of hypothalamus and pituitary gland was not revealed in head MRI. We gradually decreased hydrocortisone doses as he could not stop hydrocortisone. This was because the amount of topical steroid fluctuated according to the instability of dermatitis, and dupilumab was needed for control. Therefore, hydrocortisone was continued for another six months after his hospital admission.

## Discussion

An adverse drug reaction is defined as an appreciably harmful or unpleasant reaction caused by an intervention associated with a drug, which predicts a risk of future administration and warrants the prevention of treatment, change in dose, or cessation of the medication [4]. We reported a case of secondary adrenal insufficiency due to the termination of the topical steroid. The topical steroid was stopped after dupilumab administration. This recently developed medication for atopic dermatitis, a human monoclonal antibody that inhibits both IL-4 receptor and interleukin-13, has been reported to significantly improve moderate-to-severe atopic dermatitis [3]. The administration of newly medication led us to think of the cause as an adverse event of this medication. However, electrolyte or endocrine disorders have not been reported. The remarkable effectiveness of dupilumab made the patient’s daily life very pleasant. This led us to consider rechecking the previous medication in the patient’s history. The patient’s medicine notebook informed us about his longer-term, ultra-highly potent topical corticosteroid use.

Topical corticosteroids are classified into seven groups based on their topical steroid potency: ultra-high (I), high (II), medium to high (III), medium (IV and V), low (VI), and least potency (VII) [5]. Clobetasol propionate 0.05% used in this case is included in the ultra-highly potent steroid group [5]. Guidelines indicate that the duration of daily administration of ultra-highly potent topical corticosteroids should not exceed three weeks [6]. This patient used an ultra-highly potent topical corticosteroid for at least 18 weeks. Although topical steroids were described in the medicine notebook, the good dermal status led us to confirm the use of the topical steroid, revealing the complete cessation of topical steroid use. Dupilumab, with its remarkable effectiveness, made the use of this topical steroid completely unnecessary. We detected no abnormalities with the adrenal gland, hypothalamus, and pituitary gland such as malignant tumor, swelling, or tuberculosis in abdominal CT or head MRI in addition to the chest X-ray. Thus, we concluded that the abrupt cessation of the topical steroid caused the various symptoms related to adrenal insufficiency.

## Conclusions

If patients present some symptoms, their causes might be due to not only the side effects of newly administered drugs but also the likely effects of past medications whose use had been stopped. Moreover, even if we found that the patient’s dermal condition was good, we should confirm their use of the topical medication described in their previous medical records. Physicians need to investigate the recent history (three months prior) of the medication administered to patients, including potential topical drugs in detail.
